# Hypoxia induced mitogenic factor (HIMF) triggers angiogenesis by increasing interleukin-18 production in myoblasts

**DOI:** 10.1038/s41598-017-07952-9

**Published:** 2017-08-07

**Authors:** Chen-Ming Su, I-Ching Wang, Shan-Chi Liu, Yi Sun, Lulu Jin, Shih-Wei Wang, Hsiang-Ping Lee, Wen-Pei Tseng, Chih-Hsin Tang

**Affiliations:** 1 Department of Biomedical Sciences Laboratory, Affiliated Dongyang Hospital of Wenzhou Medical University, Dongyang, Zhejiang China; 2 Graduate Institute of Sports and Health, National Changhua University of Education, Changhua County, Taiwan; 3 Graduate Institute of Basic Medical Science, China Medical University, Taichung, Taiwan; 4 Department of Medicine, Mackay Medical College, New Taipei City, Taiwan; 5 Graduate Institute of Chinese Medicine, China Medical University, Taichung, Taiwan; 6 Department of Chinese Medicine, China Medical University Hospital, Taichung, Taiwan; 7 Department of Biotechnology, College of Health Science, Asia University, Taichung, Taiwan; 8 Department of Pharmacology, School of Medicine, China Medical University, Taichung, Taiwan

## Abstract

Inflammatory myopathy is a rare autoimmune muscle disorder. Treatment typically focuses on skeletal muscle weakness or inflammation within muscle, as well as complications of respiratory failure secondary to respiratory muscle weakness. Impaired respiratory muscle function contributes to increased dyspnea and reduced exercise capacity in pulmonary hypertension (PH), a debilitating condition that has few treatment options. The initiation and progression of PH is associated with inflammation and inflammatory cell recruitment and it is established that hypoxia-induced mitogenic factor (HIMF, also known as resistin-like molecule α), activates macrophages in PH. However, the relationship between HIMF and inflammatory myoblasts remains unclear. This study investigated the signaling pathway involved in interleukin-18 (IL-18) expression and its relationship with HIMF in cultured myoblasts. We found that HIMF increased IL-18 production in myoblasts and that secreted IL-18 promoted tube formation of the endothelial progenitor cells. We used the mouse xenograft model and the chick chorioallantoic membrane assay to further explore the role of HIMF in inflammatory myoblasts and angiogenesis *in vivo*. Thus, our study focused on the mechanism by which HIMF mediates IL-18 expression in myoblasts through angiogenesis *in vitro* and *in vivo*. Our findings provide an insight into HIMF functioning in inflammatory myoblasts.

## Introduction

Inflammation in myoblasts is associated with different conditions, such as hypoxia, strong muscle contraction, and inflammaging, a chronic low-grade inflammatory state that is associated with functional impairments, loss of strength, and myopathy^1^. Inflammatory myopathies, commonly referred to as myositis, are a rare and heterogeneous group of acquired autoimmune muscle disorders^2^. Previous research has shown that cytokines appear to play an important role in the development of inflammatory myopathy^3^.

Inflammation has also been associated with angiogenesis. Several studies have used anti-angiogenic strategies to suppress inflammation^4, 5^. Under pathological conditions, angiogenesis stimulates vascular endothelial growth factor (VEGF) expression in the smooth muscle cell phenotype^6^. Interleukin-18 (IL-18) is a pro-inflammatory cytokine that belongs to the IL-1 family, and has been identified in autoimmune disorders^7^. High levels of serum IL-18 have been observed in patients with inflammatory myopathy^8^. Moreover, vascular IL-18 has been found to correlate with tissue macrophages and increased levels are found in injured, aging vascular smooth muscle cells^9^. However, the role of IL-18 in inflammatory skeletal myoblasts remains to be elucidated.

Hypoxia-induced mitogenic factor (HIMF), also known as resistin-like molecule α, is a cysteine-rich secreted protein and pleiotropic cytokine and arises from activated macrophages of the inflammatory responses and exerts mitogenic property in inflammation and angiogenesis^10^. Previous research has reported that HIMF regulates calcium mobilization in pulmonary smooth muscle cells^11, 12^. HIMF also has significant angiogenic properties and contributes to the extension of pulmonary arterial pressure^13^. However, the regulation of HIMF in response to inflammatory myopathy remains unclear. In the present study, we describe intracellular signals showing that HIMF upregulates the expression of IL-18 through the 3-phosphoinositide-dependent protein kinase-1 (PDK1)/ phosphatidylinositol 3-kinase (PI3K)/Akt/ activator protein 1 (AP-1) signaling pathway in myoblasts. These results provide an insight into the mechanism of HIMF functioning in inflammatory myoblasts.

## Results

### HIMF induces angiogenesis through IL-18 expression in murine myoblasts

**Figure 1:**
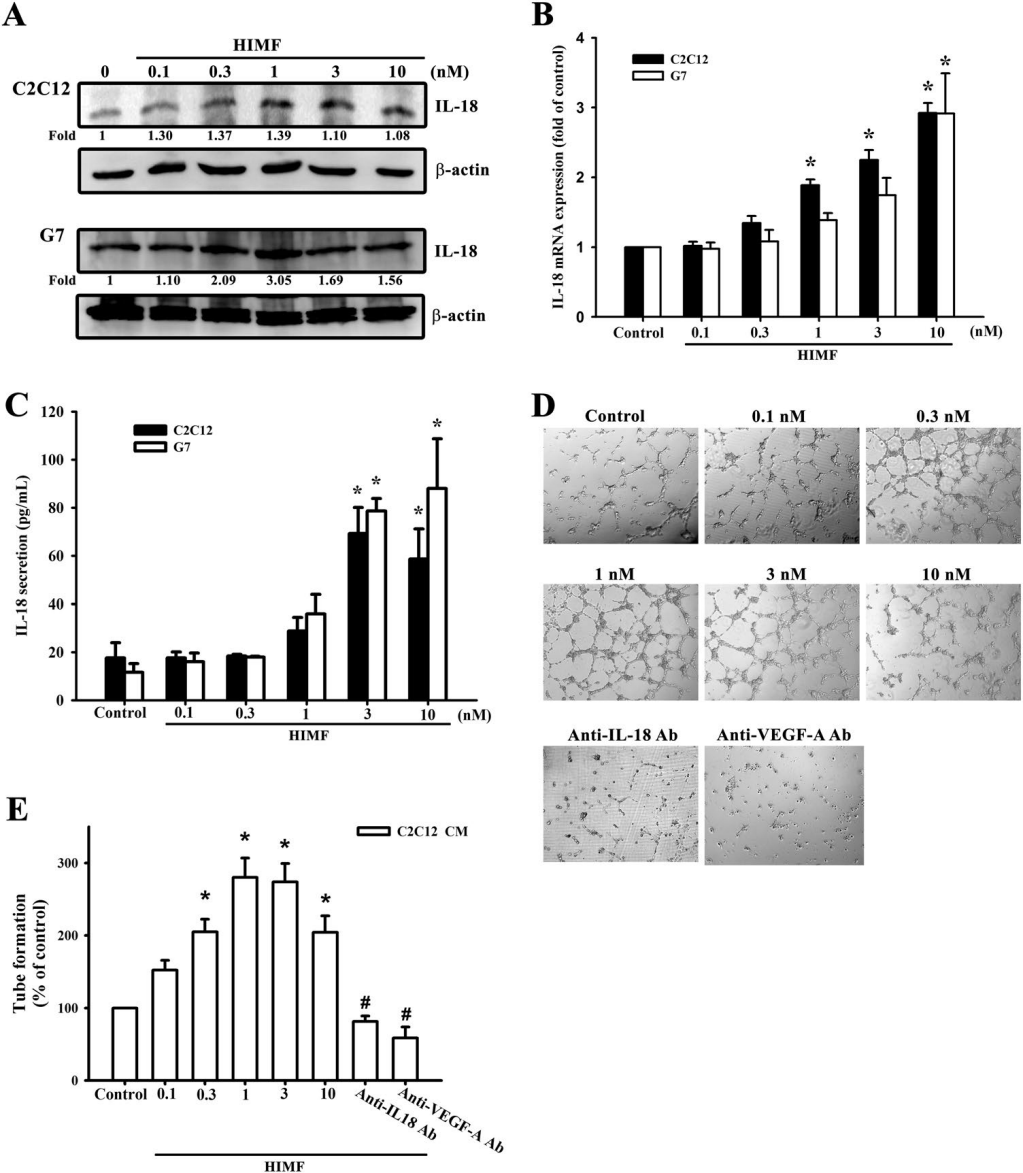
HIMF induces IL-18 expression and EPC tube formation. (**A**) HIMF induced a dose-dependent increase of IL-18 protein expression in myoblastic cell lines. (**B**) IL-18 mRNA levels were increased by HIMF in two cell lines. (**C**) Secreted IL-18 production from HIMF-treated myoblast conditioned medium was increased in a dose-dependent manner. (**D**) EPC tube formation was increased by conditioned medium from HIMF-treated myoblasts with dose-dependent manners but inhibited by anti-IL18 or anti-VEGF-A antibodies. (**E**) Quantification of tube formation. Results are expressed as the means ± SEM of four independent experiments. *p < 0.05 as compared with the control group. # p < 0.05 as compared with HIMF-treated group.

Since HIMF has been previously indicated the important role in pulmonary inflammation and angiogenesis^14^, we therefore used murine myoblasts to investigate the effects of HIMF in inflammatory responses and angiogenesis. Myoblastic cell lines (C2C12 and G7) were treated with HIMF for 24 h in dose-dependent concentrations, and an increase in IL-18 was derived from HIMF-induced protein and mRNA levels (Fig. 1A and B). Treatment of C2C12 cell line with HIMF at different time intervals induced IL-18 protein and mRNA expression in time-dependent manners (Supplementary Figure S1). These results suggested the kinetics of IL-18 release from muscle cells in response to HIMF exposure. HIMF also induced IL-18 secretion from murine myoblasts (Fig. 1C). Conditioned medium (CM) was prepared and collected from the HIMF-treated C2C12 myoblast cell line, to explore the tube formation ability of EPCs in angiogenesis. To investigate the relationship between IL-18 and angiogenesis, CM from HIMF-treated myoblasts was pretreated with anti-IL-18 or anti-VEGF antibodies for 30 min in response to EPC tube formation (Fig. 1D). The numbers of tube branches and the total tube lengths were calculated and quantified (Fig. 1E). The data indicated that HIMF induced IL-18 expression in myoblasts that could potentially correlate with angiogenesis.

### PDK1/PI3K/Akt signaling is required for the HIMF-promoted IL-18 expression

**Figure 2:**
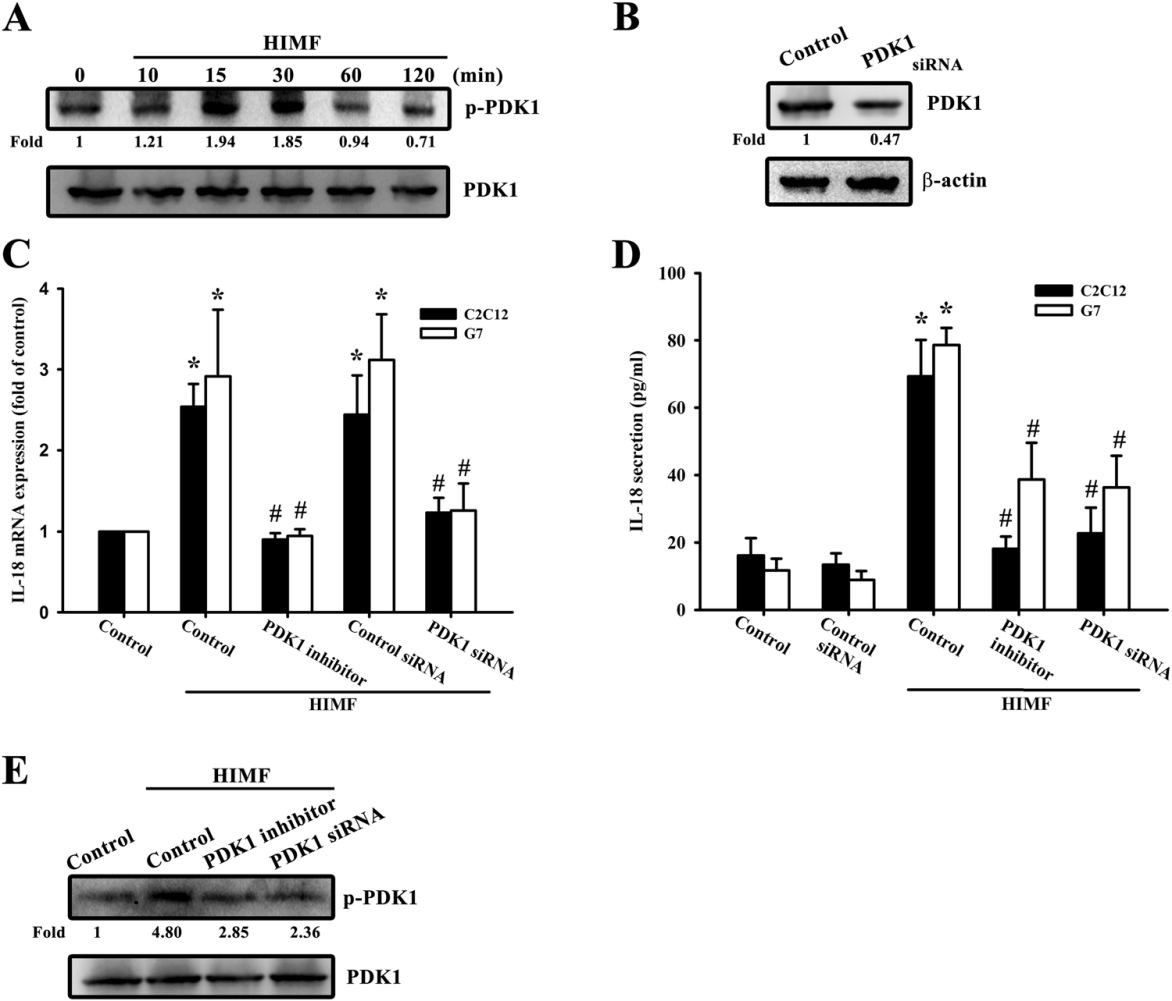
PDK1 is involved in HIMF-induced IL-18 production. (**A**) Cells were incubated with HIMF (3 nM) for indicated time intervals and cell lysates were immunoblotted with an antibody specific for phospho-PDK1. (**B**) Cells were transfected with a PDK1 siRNA for 24 h, and PDK1 protein expression was analyzed. (**C**) HIMF-induced IL-18 mRNA was inhibited by a PDK1 inhibitor or PDK1 siRNA. (**D**) Cells were pretreated for 30 min with a PDK1 inhibitor or transfected with siRNA against PDK1 for 24 h, then stimulated with HIMF for 24 h, and the conditioned media was collected and examined by ELISA. (**E**) HIMF-induced PDK1 phosphorylation was inhibited by a PDK1 inhibitor or PDK1 siRNA. Results are expressed as the means ± SEM of four independent experiments. *p < 0.05 as compared with the control group. # p < 0.05 as compared with HIMF-treated group.

**Figure 3:**
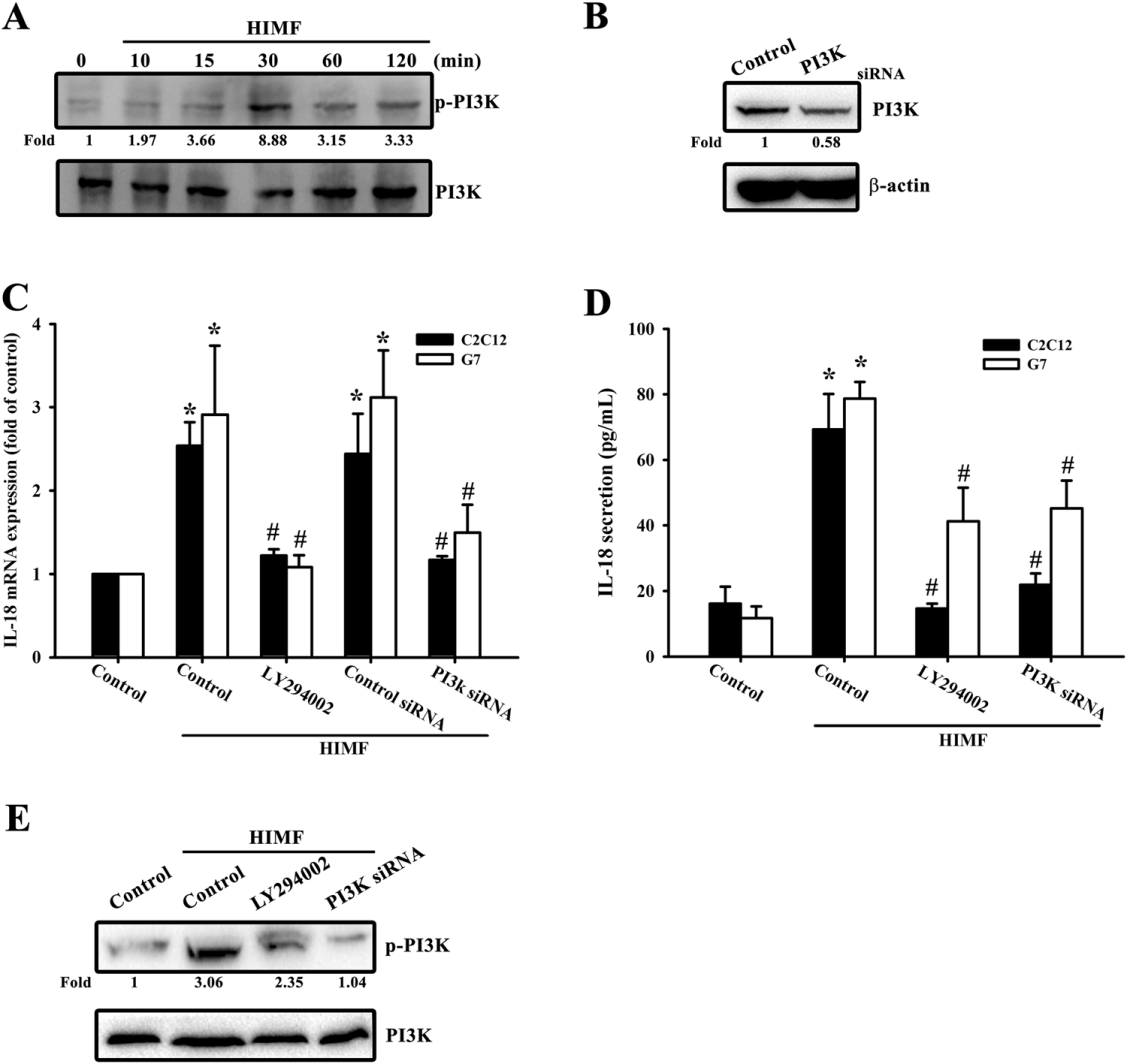
PI3K is involved in HIMF-induced IL-18 production. (**A**) Cells were incubated with HIMF (3 nM) for indicated time intervals and cell lysates were immunoblotted with an antibody specific for phospho-PI3K. (**B**) Cells were transfected with a PI3K siRNA for 24 h, and PI3K protein expression was analyzed. (**C**) HIMF-induced IL-18 mRNA levels were inhibited by LY294002 or a PI3K siRNA. (**D**) Cells were pretreated for 30 min with LY294002 or transfected with a PI3K siRNA for 24 h then stimulated with HIMF for 24 h, and the conditioned media was collected and examined by ELISA. (**E**) HIMF-induced PI3K phosphorylation was inhibited by LY294002 or PI3K siRNA. Results are expressed as the means ± SEM of four independent experiments. *p < 0.05 as compared with the control group. # p < 0.05 as compared with HIMF-treated group.

**Figure 4:**
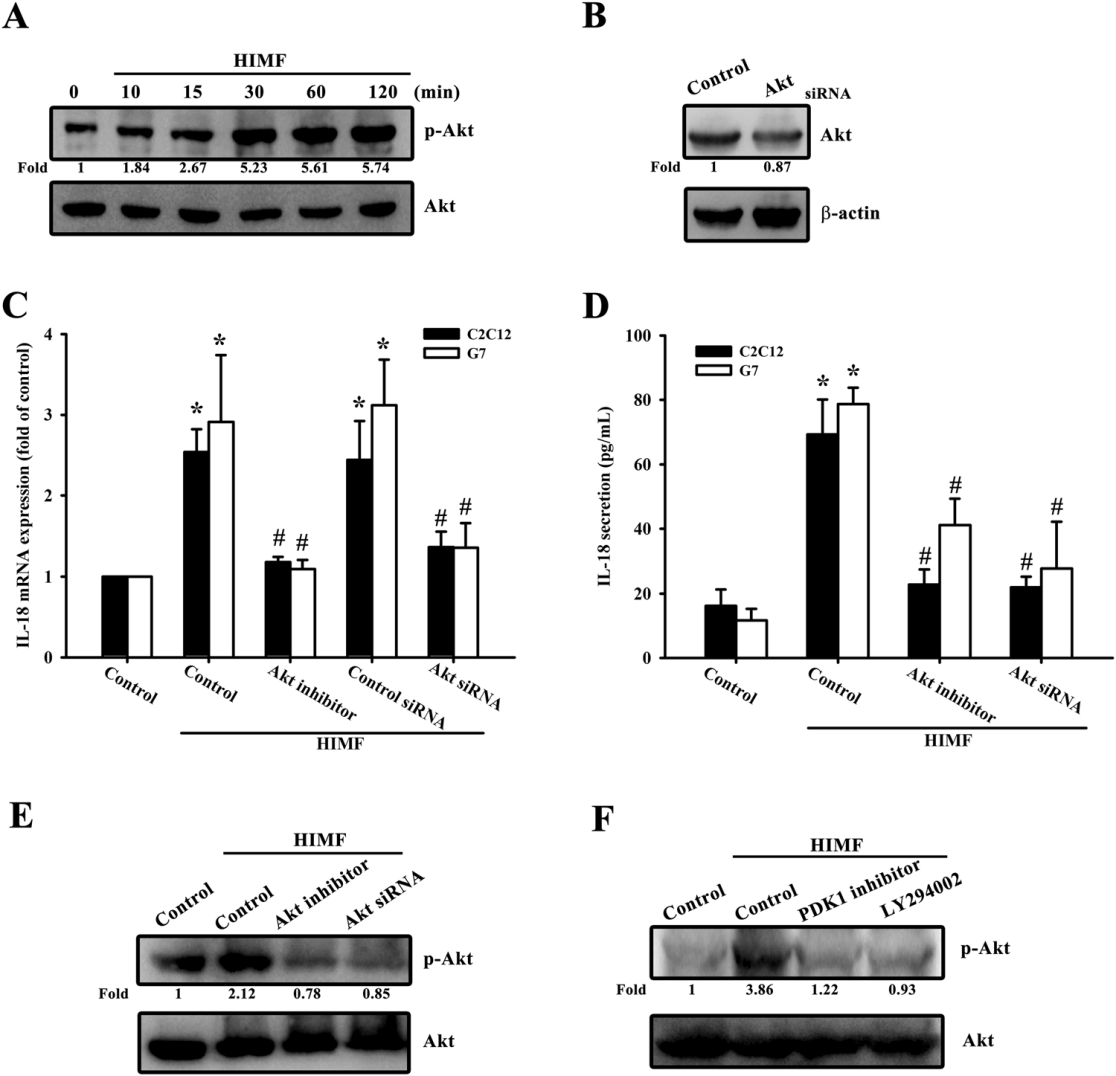
Akt is involved in HIMF-induced IL-18 production. (**A**) Cells were incubated with HIMF (3 nM) for indicated time intervals and cell lysates were immunoblotted with an antibody specific for phospho-Akt. (**B**) Cells were transfected with Akt siRNA for 24 h, and Akt protein expression was analyzed. (**C**) HIMF-induced IL-18 mRNA was inhibited by the Akt inhibitor or Akt siRNA. (**D**) Cells were pretreated for 30 min with the Akt inhibitor or transfected with Akt siRNA for 24 h, then stimulated with HIMF for 24 h, and the conditioned media was collected and examined by ELISA. (**E**) HIMF-induced Akt phosphorylation was inhibited by an Akt inhibitor or Akt siRNA. (**F**) Cells were treated with the PDK1 inhibitor or LY294002 for 30 min then stimulated with HIMF for 120 min, and phospho-Akt protein expression was analyzed. Results are expressed as the means ± SEM of four independent experiments. *p < 0.05 as compared with the control group. #p < 0.05 as compared with HIMF-treated group.

PDK1 has been reported to play a critical role in transducing inhibitory signals on eosinophil effector function^15^. We measured the signaling pathway of PDK1 phosphorylated site at Ser 241 in a time-dependent manner (Fig. 2A), and the involvement of PDK1 by using PDK1 small-interfering RNAs (siRNAs) in Western blots (Fig. 2B). Next, we detected the involvement of PDK1 in HIMF-induced IL-18 mRNA levels (Fig. 2C) and in HIMF-secreted IL-18 protein expression (Fig. 2D). We also explored the involvement of PDK1 phosphorylation in HIMF-induced IL-18 protein expression (Fig. 2E). In addition, to further confirm the signaling pathway of PDK1/PI3K, we performed an off-target assay by using constitutive active PI3K. The C2C12 cells co-transfected with a constitutive active PI3K plasmid and PDK1 siRNA for 24 h rescued PDK1 siRNA-decreased IL-18 mRNA levels, suggesting PI3K is a downstream signal of PDK1 (Supplementary Fig. S7). A recent study has demonstrated that the PI3K/Akt signaling pathway is associated with PDK1 in inflammatory diseases^16^. We continued to explore the signaling pathways of PI3K and Akt phosphorylated site at Tyr 458 and Ser 473, respectively (Figs 3A and 4A), and their involvement by using PI3K or Akt siRNA in Western blots (Figs 3B and 4B). Pretreatment of cells with PI3K or the Akt inhibitor inhibited HIMF-increased IL-18 mRNA levels and their production (Fig. 3C,D and 4C,D).We also determined that HIMF-increased Akt phosphorylation decreased in response to pretreatment with PKD1 or the PI3K inhibitor (Fig. 4E). To demonstrate the inhibition of PI3K and Akt on IL-18, we measured PI3K and Akt phosphorylation in the inhibitors and siRNAs, respectively (Figs 3E and 4F). To further confirm the signaling pathway of PI3K/Akt, C2C12 cells co-transfected with a constitutive active Akt plasmid and PI3K siRNA for 24 h rescued PI3K siRNA-decreased IL-18 mRNA levels, suggesting Akt is a downstream signal of PI3K (Supplementary Fig. S7). Above results indicate that the PDK1/PI3K/Akt signaling pathways are involved in HIMF-induced IL-18 production.

### Involvement of c-Jun in HIMF-increased IL-18 production

**Figure 5:**
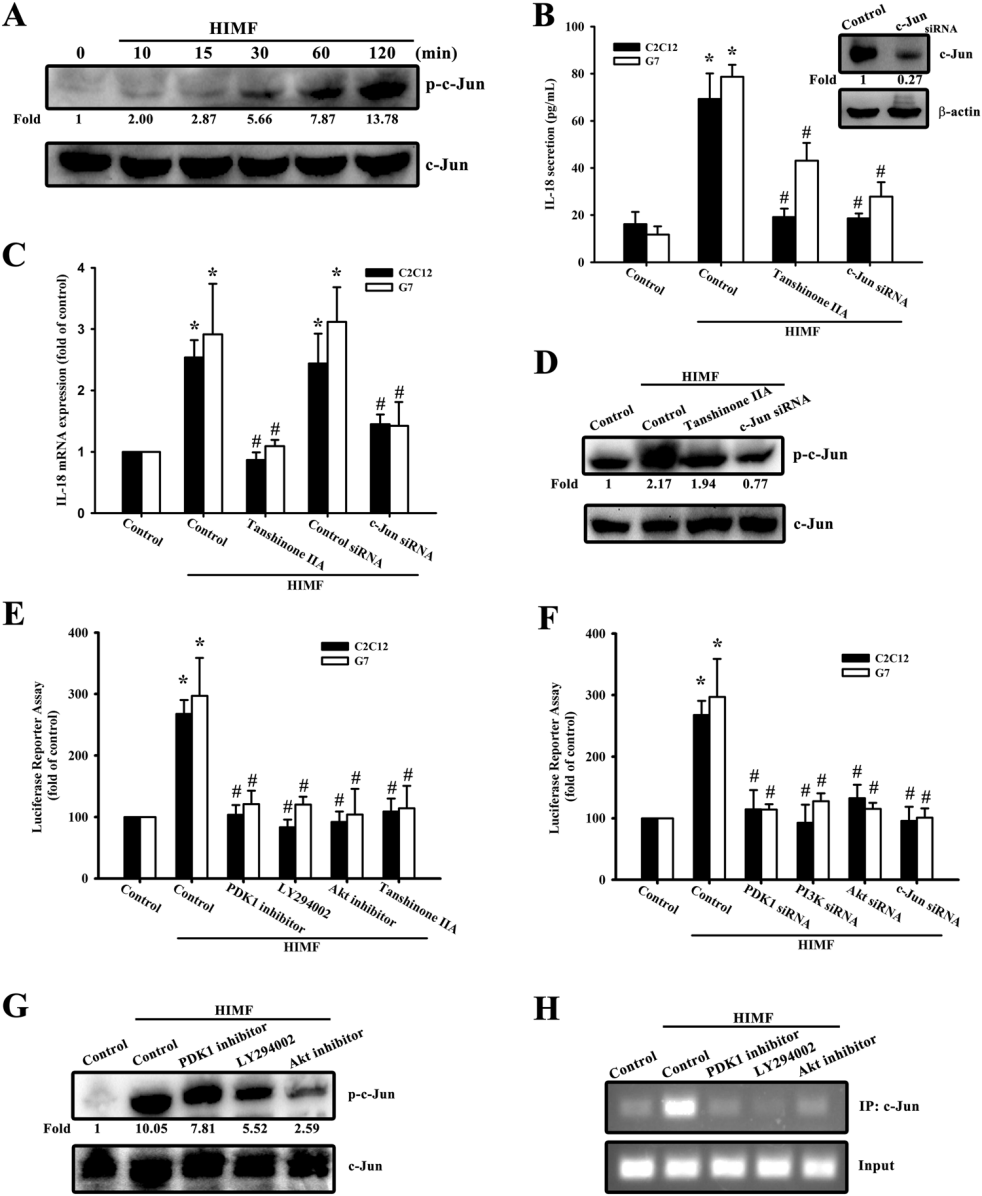
HIMF induces IL-18 production through AP-1. (**A**) Cells were incubated with HIMF (3 nM) for indicated time intervals and cell lysates were immunoblotted with an antibody specific for phospho-c-Jun. (**B**) Cells were transfected with siRNA against c-Jun for 24 h, and c-Jun protein expression was analyzed (upper panel). Cells were treated for 30 min with Tanshinone IIA or transfected with a c-Jun siRNA for 24 h, then stimulated with HIMF for 24 h, and the conditioned media was collected and examined by ELISA (lower panel). (**C**) HIMF-induced IL-18 mRNA was inhibited by Tanshinone IIA or a c-Jun siRNA. (**D**) HIMF-induced c-Jun phosphorylation was inhibited by a Tanshinone IIA or c-Jun siRNA. (**E**) Cells were treated with the PDK1 inhibitor, LY294002, an Akt inhibitor, or Tanshinone IIA for 30 min, then incubated with HIMF for 24 h. AP-1-luciferase activity was measured, and the results were normalized to β-galactosidase activity. (**F**) Cells were co-transfected with either AP-1-luciferase plasmid or siRNA against PDK1, PI3K, Akt, or c-Jun for 24 h, then stimulated with HIMF. AP-1-luciferase activity was measured, and the results were normalized to β-galactosidase activity. (**G**) Cells were treated with the PDK1 inhibitor, LY294002, or an Akt inhibitor for 30 min, then stimulated with HIMF for 120 min, and phospho-c-Jun protein expression was analyzed. (**H**) The recruitment of c-Jun to the IL-18 promoter was analyzed using the ChIP assay. Results are expressed as the means ± SEM of four independent experiments. *p < 0.05 as compared with the control group. # p < 0.05 as compared with HIMF-treated group.

To further understand the signaling mechanisms underlying HIMF-induced IL-18 production, we sought to determine which transcription factor might be implicated. AP-1 has been widely reported to be the most important transcription factor in inflammatory diseases^17^. Figure 5B showed that HIMF-increased IL-18 expression was inhibited by Tanshinone IIA which has been reported to suppress AP-1 activity with c-Jun and c-Fos DNA complex formation. To clarify the composition of AP-1 dimers being activated by HIMF exposure, we performed a co-immunoprecipitation manner followed by immunoblot analysis. After co-immunoprecipitation with anti-c-Fos antibodies for 24 h, the blotting expression of c-Jun was activated by HIMF exposure (Supplementary Fig. S2). Thus, we demonstrated c-Jun phosphorylated site at Ser 73 in a time-dependent manner (Fig. 5A), and cells transfected with c-Jun siRNA were confirmed (Fig. 5B upper panel). The role of c-Jun was involved in IL-18 secreted protein expression (Fig. 5B lower panel), mRNA levels (Fig. 5C), and protein expression (Fig. 5D) by pretreating cells with Tanshinone IIA or co-transfecting cells with c-Jun siRNA followed by HIMF treatment. To further indicate the signaling pathway of Akt/c-Jun, C2C12 cells co-transfected with a constitutive active c-Jun plasmid and Akt siRNA for 24 h rescued Akt siRNA-decreased IL-18 mRNA levels, suggesting c-Jun is a downstream signal of Akt (Supplementary Fig. S7). In order to confirm the upstream molecules engaged in HIMF-increased AP-1 signaling pathways, we used a murine AP-1 promoter-luciferase construct. HIMF markedly increased AP-1 promoter activity in myoblasts, which was reversed by pretreating cells with the PDK1 inhibitor LY294002, an Akt inhibitor, and Tanshinone IIA (Fig. 5E), and by co-transfection with PDK1, PI3K, Akt, and c-Jun siRNAs (Fig. 5F). We measured c-Jun phosphorylation through various inhibitors to confirm the upstream signals of AP-1 (Fig. 5G). In addition, we also performed IL-18 mRNA levels in which the effects of inhibitors and siRNA per se in the absence of HIMF (Supplementary Fig. S3). To demonstrate whether c-Jun could directly bind to the IL-18 target gene in the nucleus, we performed the ChIP assay and used the TFSEARCH website to examine the c-Jun binding site in the IL-18 promoter region. HIMF-induced binding of c-Jun to IL-18 was attenuated by the PDK1 inhibitor Ly294002 and the Akt inhibitor (Fig. 5H). These data suggest that HIMF-enhanced IL-18 expression requires c-Jun involvement in the signaling transduction.

### HIMF promotes IL-18-dependent angiogenesis *in vivo*

**Figure 6:**
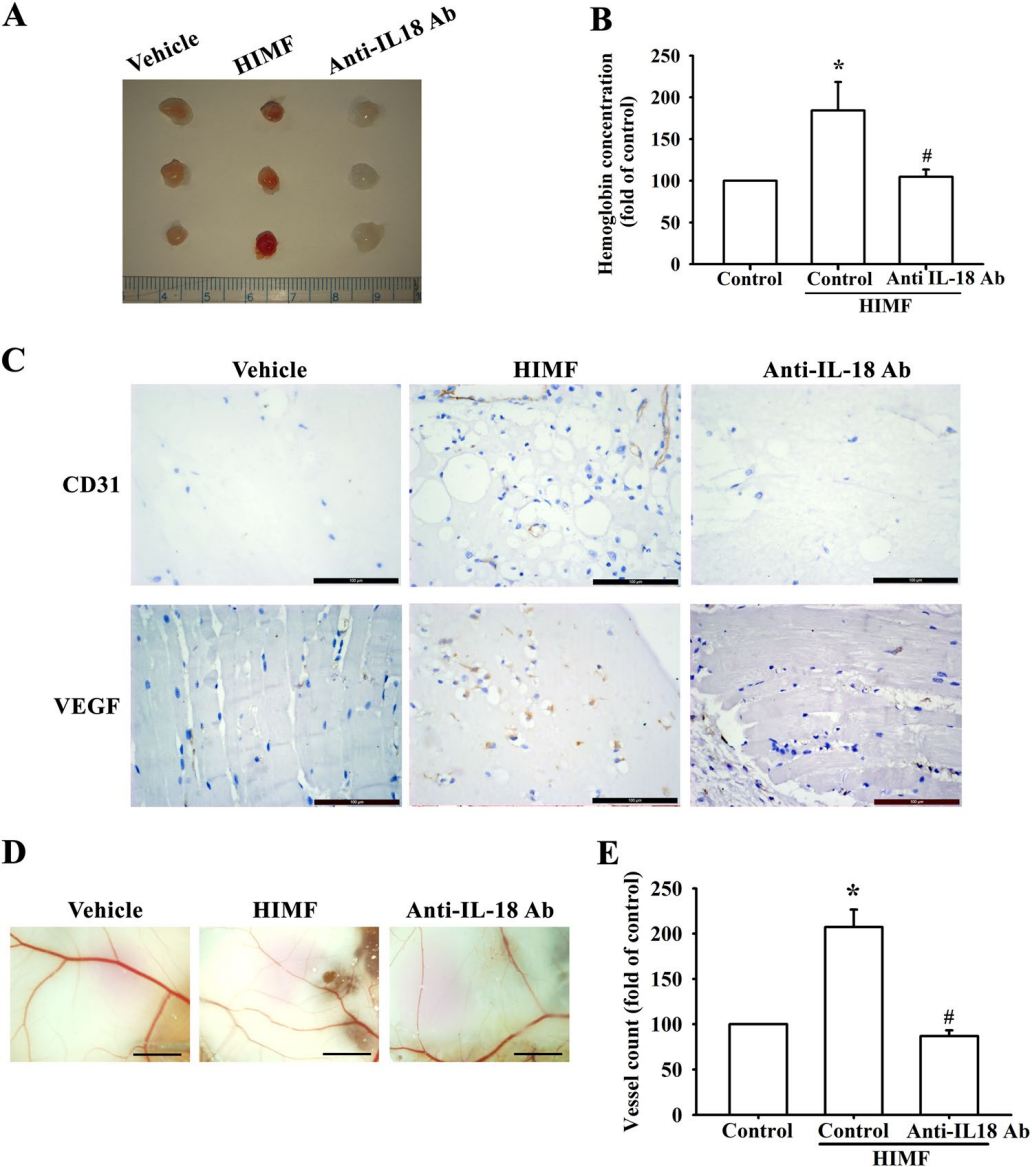
HIMF promotes VEGF expression and angiogenesis *in vivo*. (**A**) Matrigel plugs containing myoblast conditioned medium (CM) of PBS (vehicle), HIMF (10 nM), or anti-IL-18 antibody combined with HIMF were subcutaneously injected into the flanks of nude mice. After 7 days, the plugs were photographed. (**B**) Hemoglobin levels from the plugs were quantified and normalized to control group. (**C**) Specimens from the plugs were immunostained with antibodies against CD31 and VEGF. Scale bar: 100 μm. (**D**) Data from a CAM assay using 5-day-old fertilized chick embryos (6 eggs/group): PBS (vehicle), CM with HIMF (10 nM) treatment, or CM with anti-IL-18 antibody followed by HIMF treatment were resuspended in Matrigel and placed onto the CAMs, which were allowed to develop for another 3 days. The CAMs were then examined by microscopy and photographed. Scale bar: 2 mm. (**E**) CAM vessels were calculated and quantified. Results are expressed as the means ± SEM of four independent experiments. *p < 0.05 as compared with the control group. #p < 0.05 as compared with HIMF-treated group.

Our results show that secreted IL-18 expression from myoblasts CM induces EPC tube formation *in vitro*. To illustrate this angiogenesis *in vivo*, we used the mouse xenograft model. We treated myoblasts with anti-IL-18 antibody for 1 h followed by HIMF treatment. CM from HIMF-treated myoblasts was injected subcutaneously into the flanks of nude mice and incubated for 10 days. After sacrifice, the plugs were photographed and measured for hemoglobin concentration (Fig. 6A and B). The sections were immunostained with vascular markers, CD31 and VEGF (Fig. 6C). We then performed another CAM assay to determine the effect of HIMF on angiogenesis (Fig. 6D), and quantified the vessels under microscope (Fig. 6E). On the other hand, to further confirm the involvement of those signals in angiogenesis *in vivo*, CM with kinase inhibitors followed by HIMF treatment were used in Matrigel plugs and CAM assay (Supplementary Fig. S4). Our *in vivo* angiogenesis results demonstrate that HIMF-induced IL-18 secretion from myoblasts promotes angiogenesis, which might be linked to the upstream signaling pathway.

## Discussion

Inflammatory myopathy is a type of autoimmune muscle disorder. Treatment of myopathy commonly focuses on the effects in skeletal muscles in the C2C12 myoblast model^2^. A recent investigation into muscle differentiation and regeneration using the dermatomyositis autoantigen transcription intermediary factor 1γ (TIF1γ) demonstrated that premature silencing of this protein in cultured myoblasts accelerates myosin heavy chain expression (a later marker of muscle differentiation) and myoblast fusion^18^. In this study, we sought to determine how HIMF might associate with inflammatory cell infiltration in response to muscle inflammation, since it has been reported that HIMF induces endothelial smooth muscle inflammation through Th2 cell infiltration in pulmonary hypertension^19^ and increases pro-inflammatory and smooth muscle expression in lung fibroblasts^20^. However, the role of HIMF in skeletal muscle inflammation is poorly understood. Our results demonstrate that HIMF induces skeletal muscle inflammation in C2C12 and G7 cell lines, suggesting that HIMF might act as a pro-inflammatory cytokine to induce IL-18 expression related to inflammatory skeletal muscle cells.

IL-18 is a potential pro-inflammatory cytokine. Although the involvement of IL-18 has been implicated in various diseases, especially autoimmune diseases, such as allergy and systemic lupus erythematosus^7, 21^, only a few studies have demonstrated IL-18 regulation is involved in inflammatory myopathy^22^. We show that HIMF induces IL-18 expression in murine myoblasts through PDK1, which acts as a downstream effector in the PI3K/Akt/c-Jun signaling pathway and has been implicated in the regulation of neutrophil migration. These results not only provide evidence for a role of HIMF in inflammatory myopathy, but also they elucidate the mechanism of the signaling pathway.

HIMF-induced IL-18 expression through the PDK1/PI3K/Akt/c-Jun signaling pathways highlights the importance of the signaling connection between the transcription factor and target gene in the nucleus. Although there are many reports demonstrating the binding of AP-1 and IL-1β or other inflammatory cytokines, there is scant evidence on the binding of AP-1 and IL-18^23, 24^. Our findings demonstrate that HIMF augments the binding of AP-1 to the IL-18 promoter and reveal that the c-Jun binding site is located at positions −153 to −147 in the murine IL-18 promoter region. As shown in our results, HIMF-enhanced binding of AP-1 to the IL-18 promoter is antagonized by the PDK1, PI3K, or Akt inhibitors, which suggests that HIMF transcriptionally upregulates IL-18 production via the c-Jun transcription factor. Although our co-immunoprecipitation results indicate that HIMF activated the composition of c-Jun/c-Fos hetrodimers, the specific mechanism by which c-Fos involved in HIMF-mediated IL-18 production needs to be further studied by using c-Fos siRNA.

In addition to playing a role in pro-inflammation, IL-18 is a potent angiogenic mediator. VEGF, a hallmark of angiogenesis, is upregulated by pro-inflammatory cytokines or growth factors in diabetic retinopathy^25^ and therefore is involved in both inflammation and angiogenesis. Increased IL-18 expression has been found to be associated with angiogenic activity in diabetic retinopathy^26^. A previous study also demonstrated the correlation between IL-18 and VEGF in patient with diabetic retinopathy, suggesting that IL-18 may contribute to retinal angiogenesis by acting together with VEGF^27^. The role of IL-18 in angiogenesis has been further explored in recent studies into neurovascular damage and breast cancer^28, 29^. In addition, hypoxia-induced angiogenesis has recently become a critical topic for investigation not only in tumor cells but also in autoimmune diseases involving skeletal muscle^30, 31^. Here, we hypothesize that HIMF-induced IL-18 expression could be associated with angiogenesis. Since previous evidence has shown that IL18 promotes angiogenesis through the release of VEGF and other angiogenesis factor in lung cells^32^, our *in vitro* results of EPC tube formation implied that both IL-18 and VEGF-A were involved in HIMF-induced angiogenesis. Thus, the dominant angiogenesis of VEGF-A is more important than IL-18 and whether HIMF mediated rise in IL18 production caused increased angiogenesis through direct effects of IL18 on endothelial progenitor cells or through indirect effects of VEGF and other angiogenesis factors needs to be further investigated.

In conclusion, we found that HIMF increased IL-18 secretion in myoblasts and promoted EPC tube formation and angiogenesis via the PDK1/PI3K/Akt/c-Jun signaling pathway. Our evidence indicates a possible new target for drug development relating to the angiogenic mechanism by which HIMF may play a role in the pathology of inflammatory myopathies.

## Methods

### Materials

Rabbit polyclonal antibody specific for phosphate-p85, a heterodimer of PI3K, p-Akt, and p-PDK1 were purchased from Cell Signaling Technology (Danvers, MA, USA). Rabbit polyclonal antibodies specific for PDK1, PI3K, Akt, c-Jun, β-actin, and mouse polyclonal antibodies specific for IL-18 were purchased from Santa Cruz Biotechnology (Santa Cruz, CA, USA). Recombinant human HIMF was purchased from PeproTech (Rocky Hill, NJ). Neutralized monoclonal anti-IL-18 and anti-VEGF-A antibodies were both purchased from R&D Systems (Minneapolis, MN, USA). PI3K inhibitors (Ly294002), the Akt inhibitor, and c-Jun inhibitors (Tanshinone IIA) were purchased from Sigma-Aldrich (St. Louis, MO). Those siRNAs against PDK1, p85, Akt, and c-Jun were purchased from Dharmacon Research (Lafayette, CO, USA). Constitutive active plasmids of PI3K and c-Jun were gifts from Lewis Cantley (Addgene plasmid # 1399) and from Axel Behrens (Addgene plasmid # 47443), respectively. Another constitutive active plasmid of Akt was a kind gift from Dr. W.C. Huang (China Medical University, Taiwan). Dulbecco’s Modified Eagle Medium (DMEM), fetal bovine serum (FBS), and all other cell culture reagents were purchased from Gibco Life Technologies (Grand Island, NY, USA).

### Cell culture

Murine myoblast cell lines C2C12 and G7 were purchased from the American Type Culture Collection (Manassas, VA). Cell culture protocols were followed as described in previous studies^33^. C2C12 and G7 cells were cultured in DMEM supplemented with 10% FBS (Invitrogen, Carlsbad, CA) and antibiotics (100 U/mL penicillin G and 100 mg/mL streptomycin). Cultures were maintained in a humidified atmosphere of 5% CO_2_ at 37 °C.

### Human circulating EPCs

EPCs were derived from healthy donors who gave informed consent before enrollment, and ethical approval was granted by the Institutional Review Board of the Mackay Medical College (New Taipei City, Taiwan; reference number: P1000002). All research was performed in accordance with the relevant guidelines and regulations. EPCs were isolated and purified as previously described^34^. Briefly, EPCs were seeded on gelatin-coated dishes containing MV2 medium, SupplementMix (PromoCell, Heidelberg, Germany) and 20% non-heat-inactivated defined FBS (HyClone, Logan, UT, USA) and incubated for 3 days; the medium was changed every 3 days. This study’s protocol was approved by the institutional review board and the ethics committee of Human Studies Committee of China Medical University Hospital.

### Conditioned medium preparation

C2C12 (2 × 10^6^) were grown overnight in 6-well in cell culture medium. After two washes with phosphate-buffered saline (PBS), cells were incubated in 0.5% FBS in DMEM medium for 16–18 h before different stimulations. After HIMF treatment for 24 h, CM was collected for all *in vitro* and *in vivo* experiments.

### EPC tube formation

This assay was conducted as per previous protocols^34^. Briefly, EPCs (2 × 10^4^/100 µL) were resuspended in MV2 serum-free medium according to different CM and neutralized antibodies, then added to the wells with Matrigel (BD Biosciences, Bedford, MA, USA). After 6–8 h of incubation at 37 °C, EPC tube formation was assessed by microscopy, and each well was photographed at 200 × magnification under a light microscope in four random fields. The numbers of tube branches were calculated and quantified using Angiogenesis Analyzer Image J software according to previously substantial reports^35, 36^.

### Western blot analysis

Cellular lysates were prepared as described in previous studies^37^. Proteins were resolved through sodium dodecyl sulfate-polyacrylamide gel electrophoresis and transferred to Immobilon polyvinyl difluoride membranes (Millipore, Billerica, MA, USA). The blots were blocked with 4% non-fat milk for 1 h at room temperature then probed with rabbit anti-human antibodies against p-PDK1, p-PI3K, p-Akt, p-c-Jun, and mouse anti-human antibodies against IL-18, for 1 h at room temperature. After undergoing three washes, the blots were incubated with goat anti-rabbit or goat anti-mouse peroxidase-conjugated secondary antibody for 1 h at room temperature. Blots with horseradish peroxidase-labeled substrate were detected by enhanced chemiluminescence and visualized using a Fujifilm LAS-3000 chemiluminescence detection system (Fujifilm, Tokyo, Japan). All results are obtained from more than four independent experiments.

### Quantitative real-time polymerase chain reaction

Total RNA was extracted from osteoblasts using a TRIzol kit (MDBio, Taipei, Taiwan). RNA quality and yield were determined by absorbance at 260-nm measurements performed with a Nanovue Spectrophotometer (GE Healthcare, Madison, WI). Complementary DNA was synthetized from 1 μg total RNA using a Moloney Murine Leukemia Virus Reverse Transcription kit (Invitrogen), following the manufacturer’s recommendations. Quantitative real-time polymerase chain reaction (qRT-PCR) analysis was carried out with a SYBR One-Step RT-PCR Master Mix (Applied Biosystems, Foster City, CA). All target gene primers were purchased from Applied Biosystems. qPCR assays were carried out in triplicate using a StepOnePlus sequence detection system (Applied Biosystems), according to the manufacturer’s instructions. All results are obtained from six independent experiments performed in duplicate.

### Enzyme-linked immunosorbent assay

Myoblasts were cultured in 24-well culture plates. Cells were treated with HIMF and then incubated at 37 °C for 24 h. To examine the downstream signaling pathways responsive to HIMF treatment, cells were pretreated with various inhibitors for 30 minutes before the addition of HIMF (100 ng/mL). After incubation, the supernatant medium was collected and stored at −80 °C until the assay was performed. IL-18 content in the medium was assayed using an IL-18 enzyme-linked immunosorbent assay (ELISA) kit (R&D), according to the manufacturer’s instructions. All results are obtained from five independent experiments.

### Transfection and reporter gene assay

Control ON-TARGETplus small-interfering RNAs (siRNAs) and ON-TARGETplus siRNAs against PDK1, p85, Akt, and c-Jun were purchased from Dharmacon Research (Lafayette, CO, USA). Transient transfection of siRNA (0.5 nM) was carried out using Dharma FECT 1 transfection reagent (Thermo, Waltham, MA, USA) for 24 h, according to the manufacturer’s instructions.

The AP-1 luciferase plasmid is originated from the background pLuc-MCS, as 5.7 kb in length, and it contains direct seven times repeats of the transcription recognition sequences, TGACTAA. For the reporter assay as described in previous studies^38^, cells were co-transfected with AP-1 luciferase (0.5 μg) and β-galactosidase expression vector (0.5 μg) for 24 h using Lipofectamine 2000 (Invitrogen), according to the manufacturer’s protocol. All results are obtained from four independent experiments.

### Chromatin immunoprecipitation (ChIP) assay

Chromatin immunoprecipitation was performed as detailed in a previous experiment^39^. DNA pellets were immunoprecipitated by anti-c-Jun purified and extracted by phenol-chloroform, then subjected to PCR resolved by 2% agarose gel electrophoresis and visualized under UV light. Primers Forward-TCGACCCGACTCTCAACTCT and Reverse-CGTGCAGAATTTTGAGCCTG were utilized for amplification across the murine IL-18 promoter region containing c-Jun binding site.

### *In vivo* Matrigel plug assay

The Matrigel plug assay was performed as described in previous research^34^. Thirty 4-week-old male nude mice were randomized into 3 groups and subcutaneously injected with 0.15 mL Matrigel containing CM without HIMF (vehicle), CM with HIMF, or CM with anti-IL-18 antibody and HIMF, and the Matrigel:CM ratio was 1:1.5 for all groups. On day 10, the Matrigel plugs were harvested; some were fixed and embedded in paraffin and subsequently processed for immunostaining of VEGF and CD31, while others were evaluated by Drabkin’s method (Drabkin’s Reagent Kit; Sigma) to quantify hemoglobin content.

### Immunohistochemistry

Paraffin-embedded plug sections were prepared, mounted on silane-coated slides, deparaffinized in xylene, rehydrated in a graded alcohol series, and washed in deionized water. After antigen retrieval (boiling in a microwave for 30 min in 10 mM sodium citrate, pH 6.0), intrinsic peroxidase activity was blocked by incubation with 3% hydrogen peroxide. Nonspecific antibody-binding sites were blocked using 3% bovine serum albumin (BSA) in PBS. Plug sections were then incubated with the primary antibody, VEGF or CD31 (Abcam, MA, USA), at 4 °C overnight. After 3 washes in PBS, the secondary antibody (biotin-labeled goat anti-rabbit IgG) was applied for 1 h at room temperature. Staining was detected with 3,3ʹ-diaminobenzidine tetrahydrochloride (DAB) and observed under a light microscope.

### Chick chorioallantoic membrane assay

*In vivo* angiogenetic activity was determined using a chick chorioallantoic membrane (CAM) assay as described previously^34^. Briefly, 5-day-old fertilized chick embryos (6 eggs/group) were incubated at 37 °C in an 80% humidified atmosphere. On developmental day 8, CM without HIMF (vehicle), CM with HIMF, or CM with anti-IL-18 antibody and HIMF was separately re-suspended in Matrigel as per the Matrigel:CM ratio equal to 1:1.2 and placed onto the CAMs for another 3 days. The CAMs were then examined by microscopy and photographed. Angiogenesis was quantified by counting the number of blood vessel branches. All research was performed in accordance with the relevant guidelines and regulations. All animal work was performed in accordance with a protocol approved by the China Medical University’s (Taichung, Taiwan) Institutional Animal Care and Use Committee. This study’s protocol was approved by the institutional review board and the ethics committee of Human Studies Committee of China Medical University Hospital.

### Statistical analysis

Data are presented as the mean ± standard error of the mean. Statistical analysis of comparisons between the experimental groups and control was performed by using the Student’s t test. For Statistical comparisons of multi-groups, we used a two-way analysis-of-variance with statistical significance (experiments versus control) and negative control as the fixed factors. Main and interaction effects were tested at a critical level of α = 0.05. In all cases, p < 0.05 was considered significant.

## Electronic supplementary material

Dataset 1
