# Supplementary material for: Hypoxia induced mitogenic factor (HIMF) triggers angiogenesis by increasing interleukin-18 production in myoblasts
Source: Sci Rep. 2017 Aug 7;7:7393. doi: 10.1038/s41598-017-07952-9 (PMC5547156; doi:10.1038/s41598-017-07952-9)
Supplement: Supplementary file 1 — Dataset 1 [file 41598_2017_7952_MOESM1_ESM.doc]

**Hypoxia induced mitogenic factor (HIMF) triggers angiogenesis by increasing interleukin-18 production in myoblasts**

**Chen-Ming Su^1^, I-Ching Wang^2^, Shan-Chi Liu^3^, Yi Sun^1^, Lulu Jin^1^, Shih-Wei Wang^4^, Hsiang-Ping Lee^5,6^, Wen-Pei Tseng^2*^, and Chih-Hsin Tang^3,7,8^***

^1^Department of Biomedical Sciences Laboratory, Affiliated Dongyang Hospital of Wenzhou Medical University, Dongyang, Zhejiang, China

^2^Graduate Institute of Sports and Health, National Changhua University of Education, Changhua County, Taiwan

^3^Graduate Institute of Basic Medical Science, China Medical University, Taichung Taiwan

^4^Department of Medicine, Mackay Medical College, New Taipei City, Taiwan

^5^Graduate Institute of Chinese Medicine, China Medical University, Taichung, Taiwan

^6^Department of Chinese Medicine, China Medical University Hospital, Taichung, Taiwan

^7^Department of Biotechnology, College of Health Science, Asia University, Taichung, Taiwan

^8^Department of Pharmacology, School of Medicine, China Medical University, Taichung, Taiwan

***Corresponding author**

Chih-Hsin, Tang PhD

Graduate Institute of Basic Medical Science, China Medical University

No. 91, Hsueh-Shih Road, Taichung, Taiwan

Tel: (886) 4-22052121 Ext. 7726. Fax: (886) 4-22333641.

E-mail: [chtang@mail.cmu.edu.tw](mailto:wenmei@ha.mc.ntu.edu.tw)

***Co-corresponding author**

Wen-Pei, Tseng PhD

Graduate Institute of Sports and Health, National Changhua University of Education, Changhua County, Taiwan

No.1, Jin-De Road, Changhua City, Taiwan

Tel: (886) 4-7232105

E-mail: twp@cc.ncue.edu.tw

**Supplementary Figures**

**
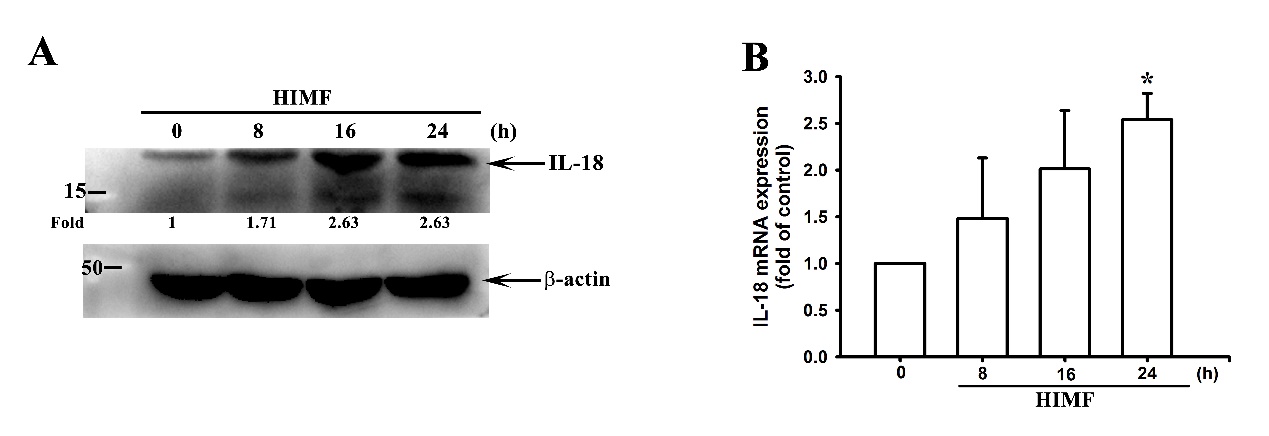
**

**Figure S1. The kinetics of IL-18 release from muscle cells in response to HIMF exposure. A.** HIMF induced IL-18 protein and **B.** mRNA expression in myoblasts in a time-dependent manner. * *p* < 0.05 as compared with the control group.

**
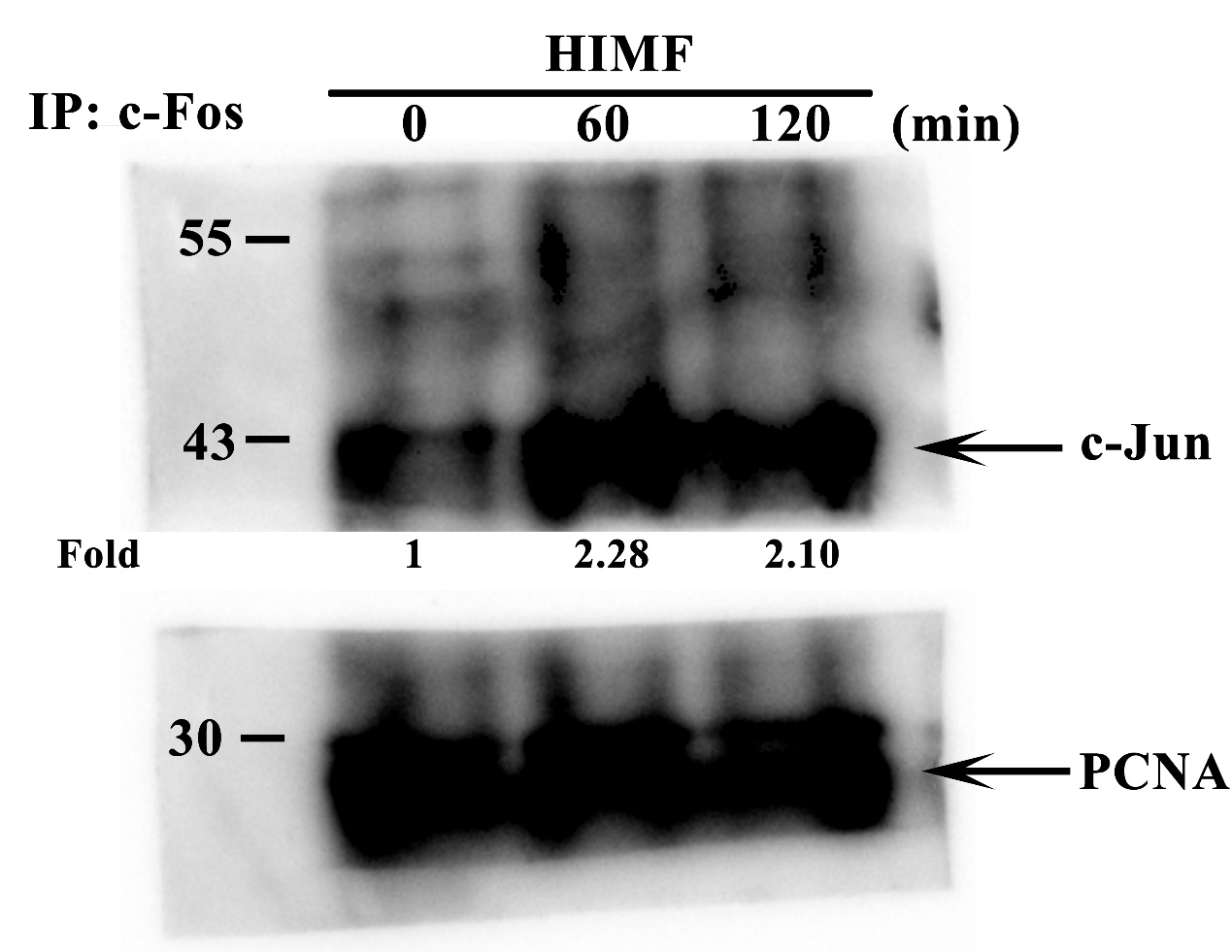
**

**Figure S2.** **The composition of AP-1 dimers is activated by HIMF exposure.** Equal amounts of cell lysates treated with HIMF were immunoprecipitated with anti-c-Fos antibody, followed by Western blot analysis with anti-c-Jun antibody.

**
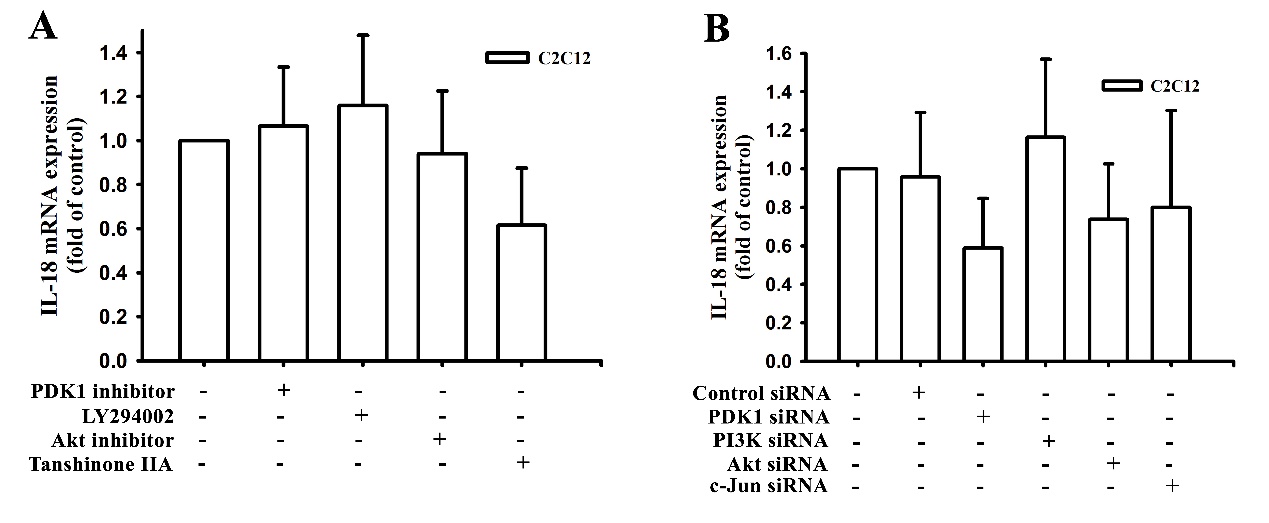
**

**Figure S3. The effects of IL-18 in the absence of HIMF by signaling pathway, inhibitors and gene knockdown with siRNAs in the myoblasts. A.** Cells were pretreated with the PDK1 inhibitor, LY294002, Akt inhibitor, or Tanshinone IIA for 30 min, and IL-18 mRNA level was analyzed. **B.** Cells were transfected with siRNA against PDK1, PI3K, Akt, or c-Jun for 24h, and IL-18 mRNA level was analyzed.

**
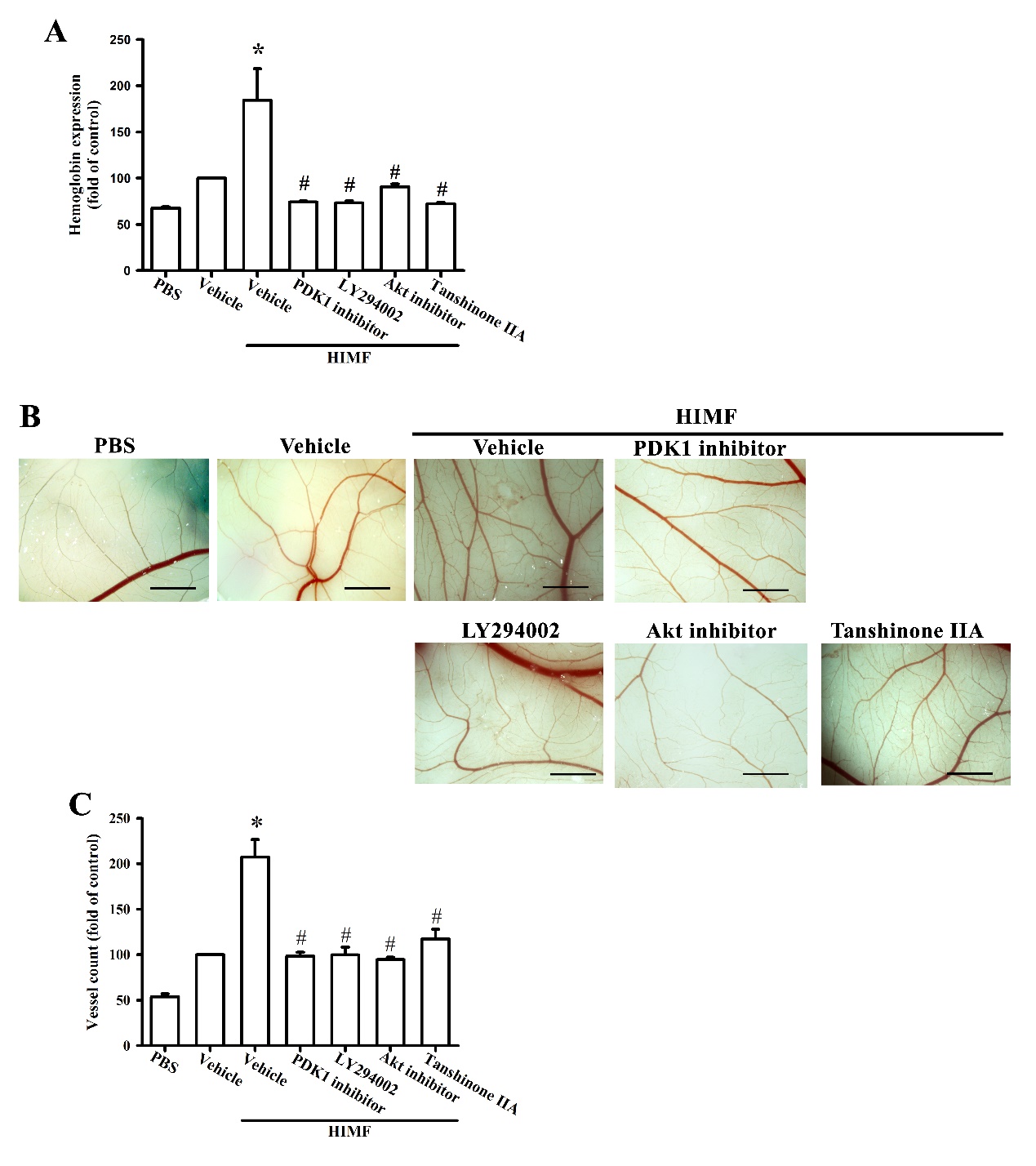
**

**Figure S4.** ***In vivo* results of angiogenesis using Matrigel plugs and chick chorioallantoic membrane (CAM) assay.** **A.** Matrigel plugs containing PBS, myoblast conditioned medium (CM) with control (vehicle), HIMF, PDK1 inhibitor, LY294002, Akt inhibitor, or Tanshinone IIA followed by HIMF treatment were subcutaneously injected into the flanks of nude mice. After 7 days, the plugs were removed and analyzed the hemoglobin levels which were normalized to vehicle group (n = 8). **B.** Data from a CAM assay using 5-day-old fertilized chick embryos (6 eggs/group): PBS, CM with control (vehicle), HIMF, PDK1 inhibitor, LY294002, Akt inhibitor, or Tanshinone IIA followed by HIMF treatment were resuspended in Matrigel and placed onto the CAMs, which were allowed to develop for another 3 days. The CAMs were then examined by microscopy and photographed. Scale bar: 2 mm. **C.** CAM vessels were calculated and quantified. Results are expressed as the means ± SEM of six independent experiments. * *p* < 0.05 as compared with the vehicle group. # *p* < 0.05 as compared with the HIMF-treated group.





**Figure S5. Uncropped blots for Western blot.**

**A.** Full-length immunoblots of Figure 1A.

**B.** Full-length immunoblots of Figure 2A.

**C.** Full-length immunoblots of Figure 3A.

**D.** Full-length immunoblots of Figure 4A.

**E.** Full-length immunoblots of Figure 5A.

**F.** Full-length immunoblots of Figure 2B.

**G.** Full-length immunoblots of Figure 3B.


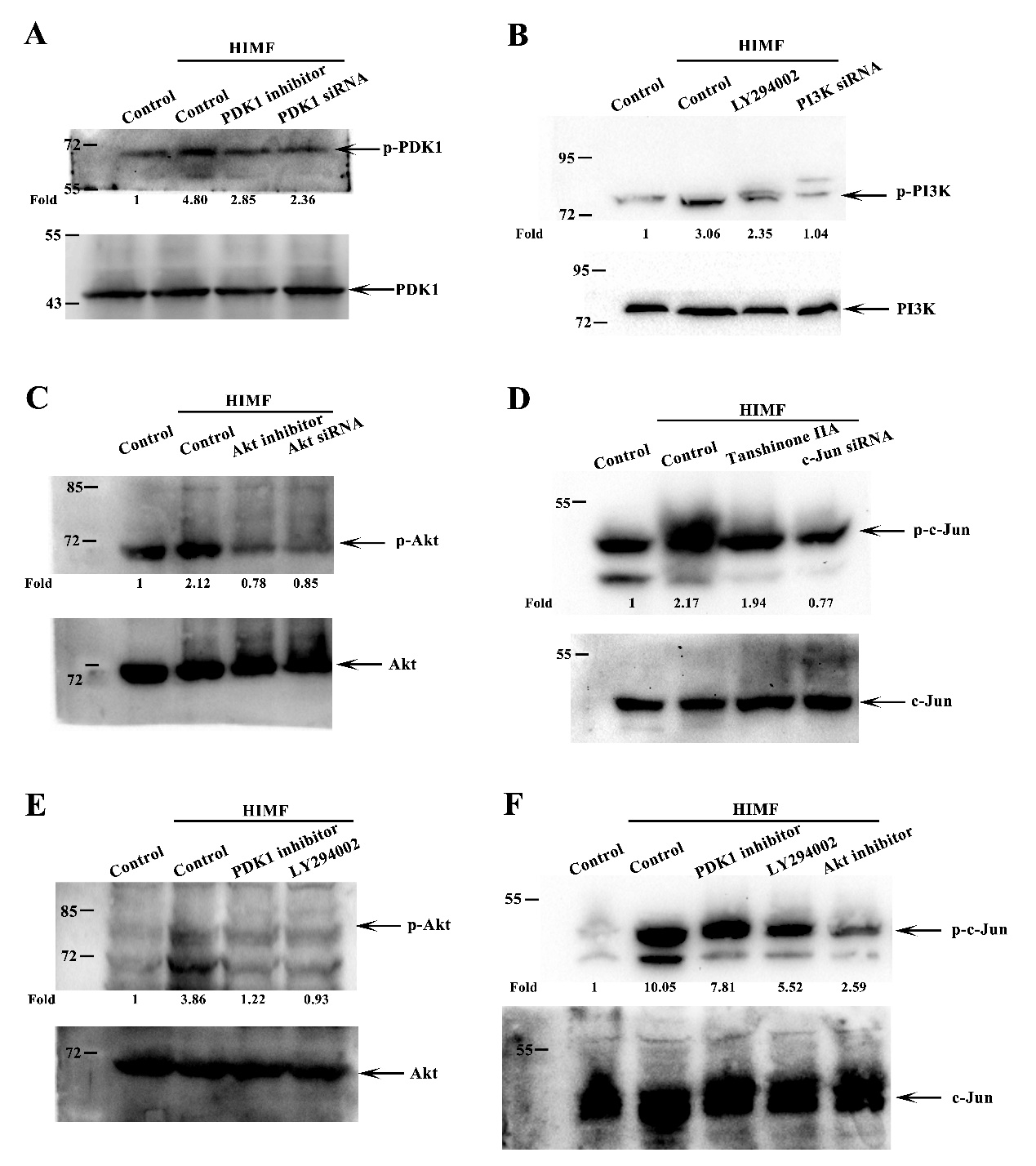


**Figure S6. Uncropped blots for Western blot.**

**A.** Full-length immunoblots of Figure 2E.

**B.** Full-length immunoblots of Figure 3E.

**C.** Full-length immunoblots of Figure 4E.

**D.** Full-length immunoblots of Figure 5D.

**E.** Full-length immunoblots of Figure 4F.

**F.** Full-length immunoblots of Figure 5G.


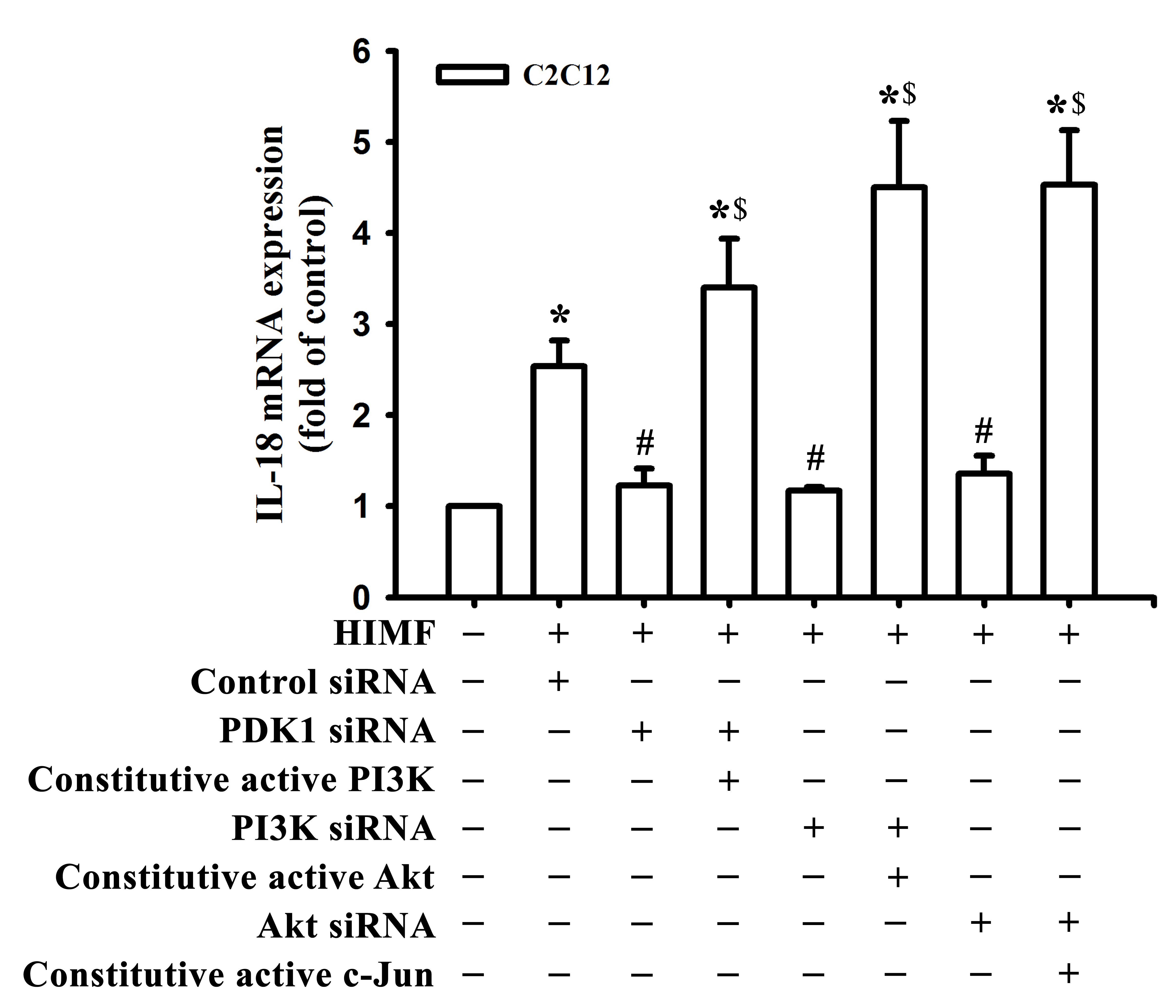


**Figure S7. HIMF increased IL18 expression in C2C12 myoblast via the PDK1/PI3K/Akt/cJun signaling pathway.** Cells were co-transfected with PDK1 siRNA and constitutive active PI3K, PI3K siRNA and constitutive active Akt, and Akt siRNA and constitutive active c-Jun for signaling pathway of PDK1→PI3K, PI3K → Akt, and Akt → c-Jun, respectively. IL-18 mRNA level was analyzed. Results are expressed as the means ± SEM of six independent experiments. * *p* < 0.05 as compared with the control group. # *p* < 0.05 as compared with the HIMF-treated and control siRNA-transfected group. $ *p* < 0.05 as compared with PDK1, PI3K, or Akt siRNA-transfected group.
